# The low-grade Inflammation Score (INFLA-Score) as a predictor of overall survival in prostate cancer: a UK biobank cohort study

**DOI:** 10.1186/s12894-026-02078-5

**Published:** 2026-05-08

**Authors:** Suxin Jiang, Xiaojie Zheng, Hao Qiu, Ning Tao, Hengqing An

**Affiliations:** 1 College of Public Health, Xinjiang Medical University, Urumqi, Xinjiang Uygur Autonomous Region China; 2 Science and Technology Management Office, Teaching and Research Administration Department, The First Affiliated Hospital of Xinjiang Medical University, Urumqi, Xinjiang Uygur Autonomous Region China; 3 Department of Urology, The First Affiliated Hospital of Xinjiang Medical University, Urumqi, Xinjiang Uygur Autonomous Region China

**Keywords:** INFLA-score, Inflammatory markers, Prostate cancer, Prognosis, UK biobank

## Abstract

**Objective:**

Inflammation plays a crucial role in the progression and prognosis of prostate cancer (PCa). The aim of this study was to investigate the association of the low-grade inflammation score (INFLA-score) with overall survival in patients with prostate cancer.

**Methods:**

Utilizing data from the UK Biobank, we included 13,166 men diagnosed with PCa. The predictive accuracy of systemic inflammatory biomarkers for overall survival was assessed and compared using the C-statistic. Survival curves were plotted using the Kaplan-Meier method and differences in in overall survival between groups were compared by the log-rank test. Restricted cubic spline (RCS) curves were used to explore the relationship between biomarkers and survival. Independent prognostic biomarkers associated with overall survival (OS) were determined using multivariable Cox proportional hazards regression analysis.

**Results:**

The INFLA-score demonstrated the highest predictive accuracy for overall survival among all evaluated biomarkers, with a C-statistic of 0.556 (0.544,0.567). Patients with a high INFLA-score had significantly worse overall survival (shorter survival time) than those with a low INFLA-score (33.76% vs. 66.24%; log-rank *p* < 0.001). RCS analysis revealed a significant dose-response relationship between the INFLA-score and survival in PCa patients. After adjusting for potential confounders, a high INFLA-score remained an independent predictor of poor OS (HR = 1.24, 95% CI: 1.15–1.33, *p* < 0.001).

**Conclusions:**

The INFLA-score was independently associated with overall survival of PCa patients. As an easily obtainable and integrative measure of low-grade inflammation, it shows significant promise for clinical application in risk stratification and survival prediction.

**Supplementary Information:**

The online version contains supplementary material available at 10.1186/s12894-026-02078-5.

## Introduction

Prostate cancer (PCa) is one of the malignant tumors with the highest morbidity and mortality rates worldwide, severely affecting the health and quality of life of middle-aged and older men. According to the Global Cancer Statistics 2020, prostate cancer is the fourth most commonly diagnosed cancer and the eighth leading cause of cancer-related deaths in men worldwide [1]. The global incidence of PCa is projected to rise to approximately 1.7 million new cases and 499,000 deaths by 2030, with population aging being a key driver of this increase [2]. Despite recent advances in PCa treatments, the improvement in long-term survival of patients is still very limited, reflecting the inadequacy of current prognostic assessment systems. Therefore, the search for simple, inexpensive, and effective biomarkers has become an urgent need to improve PCa survival assessment and individualized treatment.

PCa is a multifactorial systemic disease. Currently, Prostate-Specific Antigen (PSA) is still the most commonly used non-invasive test for screening and efficacy assessment of PCa. PSA is highly specific for prostate tissue, but its diagnostic sensitivity for PCa is limited [3]. Therefore, the development of new biomarkers is gradually showing potential [4–6]. In recent years, several studies have shown that chronic inflammatory states are closely associated with the development and progression of malignant tumors. Several epidemiological studies have emphasized that chronic inflammation caused by hormonal imbalance, chemical exposure, radiation, or viral infection are important risk factors for prostate cancer [7]. Within the tumor microenvironment, infiltrating immune cells (e.g., macrophages, T cells) and their secreted inflammatory mediators (e.g., cytokines, chemokines) activate and sustain local inflammatory responses. These responses regulate the behavior of tumor and stromal cells via autocrine and paracrine signaling, thereby promoting key malignant processes such as sustained proliferation, invasion, and metastasis [8]. This interaction not only exacerbates tumor progression, but also significantly correlates with poor patient prognosis and decreased quality of survival. As a complex ecosystem composed mainly of immune cells, fibroblasts and extracellular matrix, the tumor microenvironment has become an important source of potential prognostic biomarkers for PCa [9]. Therefore, inflammation-related indicators show significant value in the diagnosis, treatment and prognosis prediction of cancer.

Among the various types of systemic inflammatory biomarkers, composite indicators constructed on the basis of peripheral blood cell parameters have demonstrated significant prognostic value in a variety of cancers, including PCa [10–12]. Current research has primarily focused on single indicators, such as the neutrophil-to-lymphocyte ratio (NLR). Although widely used, these reflect only a single dimension of the inflammation-immune balance [13–15]. In contrast, the low-grade inflammation score (INFLA-score) is an emerging composite index that integrates four parameters—C-reactive protein (CRP), platelet count, white blood cell count (WBC), and the neutrophil-to-lymphocyte ratio (NLR)—and theoretically provides a more comprehensive assessment of systemic inflammation [16]. This score has shown significant associations with disease risk in cardiovascular studies [17]and certain malignancies [18]; however, its value in prognostic assessment for prostate cancer, particularly its comparative advantage over existing indicators, remains underexplored and unclear. Therefore, this study aimed to systematically evaluate and compare the prognostic performance of existing systemic inflammation biomarkers to identify the most optimal one for PCa. We placed particular emphasis on investigating the potential of the INFLA-score as a novel prognostic biomarker. Our analysis focuses on the association between the INFLA-score and overall survival (OS) to assess its value in stratifying survival risk among PCa patients.

## Materials and methods

### Study design and population

This study utilized data from the UK Biobank, a large-scale prospective cohort that recruited approximately 500,000 individuals aged 40–69 between 2006 and 2010 (https://www.ukbiobank.ac.uk). At recruitment, comprehensive baseline data were collected, including demographics, lifestyle, occupational information, biological samples, and medical conditions. Incident prostate cancer cases were identified through linkage to national cancer registries (UKB Field ID: 40006). Due to varying data release schedules, the final follow-up end dates were 31 October 2022 for England, 31 May 2022 for Wales, and 31 August 2022 for Scotland. Participants without a clinical event (prostate cancer diagnosis, death, or loss to follow-up) by their region-specific date were right-censored. The analytical cohort was restricted to men diagnosed with prostate cancer (ICD-10 code: C61) who had complete data on all hematological parameters. The UK Biobank has received ethical approval from the North West Multi-centre Research Ethics Committee and operates as a research tissue bank; therefore, no separate ethical approval was required for this study.

### Definition of exposure and covariates

The hematologic parameters assessed in this study included white blood count, neutrophils, lymphocytes, platelets, CRP, high-density lipoprotein (HDL) cholesterol, glucose, and triglycerides. The primary outcome was overall survival (OS), defined as the time from the date of prostate cancer diagnosis to the date of death from any cause, with participants alive at the end of follow-up being right-censored. Covariates were selected based on their established clinical or statistical relevance to prostate cancer outcomes. These encompassed age, race, years of education, the Townsend Deprivation Index, body mass index (BMI), physical activity level (categorized as Low, Moderate, High), smoking status (never, previous, current), alcohol consumption (never, previous, current), a history of prostate diseases (including prostate hyperplasia and inflammatory prostate disease), family history of PCa, fasting glucose, testosterone, hypertension, diabetes, cardiovascular disease, and a history of prostate-specific antigen (PSA) testing.

### Calculation of inflammatory biomarkers

This study incorporated multiple systemic inflammatory biomarkers, including the Neutrophil-to-Lymphocyte Ratio (NLR), Lymphocyte-to-High-density lipoprotein Ratio (LHR), Systemic Immune-inflammation Index (SII), Inflammatory Burden Index (IBI), C-reactive protein-Triglyceride-Glucose Index (CTI), and the low-grade inflammation score (INFLA-score).

The formulas for calculating these indices are as follows:

$$\begin{aligned} \mathrm{NLR}=&\:\text{Neutrophil count}\left(10^9/ \mathrm{L}\right)\\&/\: \text{Lymphocyte count}\left(10^9/ \mathrm{L}\right) \end{aligned}$$

$$\begin{aligned} \mathrm{LHR}=&\:\text{Lymphocyte count}\left(10^9/ \mathrm{L}\right)\\&\:/\,\text{High-density lipoprotein cholesterol}\left(\mathrm{mmol/L}\right) \end{aligned}$$

$$\begin{aligned} \mathrm{SII}=\:&\left(\text{Platelet count}\left(10^9/ \mathrm{L}\right)\times \text{Neutrophil count}\left(10^9/\mathrm{L}\right)\right)\\& / \:\text{Lymphocyte count}\left(10^9/ \mathrm{L}\right) \end{aligned}$$

$$\begin{aligned} \mathrm{IBI}=&\:\text{C-reactive protein}\,\left(\mathrm{mg/L}\right)\\&\times\left(\text{Neutrophil count}\left(10^9/ \mathrm{L}\right) \right. \\& \left. / \: \text{Lymphocyte count}\left(10^9/ \mathrm{L}\right)\right) \end{aligned}$$

$$\begin{aligned} \mathrm{CTI}=&\:0.412\times\,\mathrm{ln}\left(\mathrm{CRP}\left[\mathrm{mg/L}\right]\right)\\&+\mathrm{ln}\left(\mathrm{Triglycerides}\left[\mathrm{mg/dL}\right] \right. \\& \left. \times\,\text{Fasting glucose}\left[\mathrm{mg/dL}\right]/2\right) \end{aligned}$$

The INFLA-score consists of four components: CRP, WBC, platelet count, and NLR, which is considered to be a composite indicator of low-grade inflammation in the body. To calculate the INFLA-score, all components in the highest decile (7 to 10) were assigned a value of + 1 to + 4, while biomarker levels in the lowest decile (1 to 4) were assigned a value of − 4 to − 1. INFLA-score ranged from − 16 to + 16, with higher scores indicating higher levels of low-grade chronic inflammation [19].

## Statistical analysis

In this study, missing covariate data were handled using multiple imputation. Categorical variables were presented as numbers (percentages) and compared using the χ² test. Continuous variables were summarized as mean ± standard deviation or median (interquartile range) based on their distribution, and compared using the Student’s t-test or Mann-Whitney U test, respectively. The predictive accuracy of systemic inflammatory biomarkers for overall survival was evaluated using the C-statistic. The optimal cutoff of the INFLA-score for predicting overall survival was determined using the maximally selected log-rank statistic. The association between the INFLA-score and overall survival was visualized using restricted cubic spline (RCS) curves. Overall survival differences between groups were assessed with the log-rank test and plotted using Kaplan-Meier curves. Independent factors associated with OS were identified using multivariable Cox proportional hazards regression, with significance evaluated using the Wald test. All analyses were performed using R software (version 4.5.1) and a two-sided *p* < 0.05 was considered statistically significant.

## Results

### Baseline characteristics of PCa participants

Patients with case PCa were screened from the UKB database. After excluding patients with missing serologic data, a total of 13,166 PCa patients with a mean age of 63.0 (59.0, 66.0) years were enrolled in the study. The demographic and clinicopathological characteristics of the patients are summarized in Table 1.

**Table 1:** Baseline characteristics of the prostate cancer cohort

Characteristic	Total (*N* = 13,166)^1^
White blood cells (10⁹/L), Median [Q1, Q3]	6.7 [5.7, 7.9]
Platelets (10⁹/L), Median [Q1, Q3]	233.0 [199.5, 268.0]
C-reactive protein (mg/L), Median [Q1, Q3]	1.3 [0.7, 2.5]
Neutrophils (10⁹/L), Median [Q1, Q3]	4.1 [3.4, 5.0]
Lymphocytes (10⁹/L), Median [Q1, Q3]	1.8 [1.4, 2.2]
Age (years), Median [Q1, Q3]	63.0 [59.0, 66.0]
Townsend deprivation index, Median [Q1, Q3]	-2.4 [-3.8, -0.1]
Ethnicity, n (%)	
White	12,633.0 (96.0%)
Black	279.0 (2.1%)
Asian	131.0 (1.0%)
Other	123.0 (0.9%)
Education (years), Median [Q1, Q3]	17.0 [15.0, 20.0]
Smoking status, n (%)	
Never	6,258.0 (47.5%)
Former	5,738.0 (43.6%)
Current	1,170.0 (8.9%)
Alcohol consumption, n (%)	
Never	313.0 (2.4%)
Occasional	402.0 (3.0%)
Regular	12,451.0 (94.6%)
Body mass index (kg/m²), Median [Q1, Q3]	27.2 [25.0, 29.8]
Physical activity, n (%)	
Low	2,336.0 (17.7%)
Moderate	5,254.0 (39.9%)
High	5,576.0 (42.1%)
History of prostate disease, n (%)	1,867.0 (14.2%)
Family history of prostate cancer, n (%)	1,465.0 (11.1%)
Testosterone (nmol/L), Median [Q1, Q3]	11.5 [9.3, 13.9]
Previous PSA screening, n (%)	7,103.0 (53.9%)
Diabetes mellitus, n (%)	838.0 (6.4%)
Hypertension, n (%)	5,237.0 (39.8%)
Cardiovascular disease, n (%)	1,049.0 (8.0%)

^1^Data presented as median [interquartile range] or number (%)

### INFLA-score demonstrates best predictive value for OS among systemic inflammation biomarkers

Systemic inflammation is characterized by a reduced ratio of pro-inflammatory parameters (neutrophils, platelets and CRP) to anti-inflammatory parameters (lymphocytes and albumin). We evaluated each of these five serum pro- and anti-inflammatory parameters in combination. Ultimately, we identified a representative set of six systemic inflammatory biomarkers (Table 2), which included both previously reported and novel composite indices. We found that among these systemic inflammatory biomarkers, INFLA-score had the highest C-statistic for predicting all-cause mortality in PCa patients, at 0.556 (0.554, 0.567). After adjustment for multiple comparisons using the false discovery rate method, the INFLA-score remained significantly superior to LHR (ΔC-index = 0.029, q = 0.001), SII (ΔC-index = 0.031, q = 0.001), and NLR (ΔC-index = 0.036, q < 0.001), while no significant differences were observed compared to CTI (ΔC-index = 0.011, q = 0.234) and IBI (ΔC-index = 0.004, q = 0.667).These results suggest that the INFLA-score is the most discriminatory systemic inflammatory biomarker for stratifying survival risk in PCa patients. Therefore, we further evaluated its potential as a prognostic biomarker for this population.

**Table 2:** Comparative analysis of the discrimination of each systemic inflammation-related biomarkers for all-cause mortality in prostate cancer

Discrimination ability	C-statistic			FDR q
	Difference	Difference	*P* value	
INFLA score	0.556(0.544,0.567)	Ref		
CTI	0.544(0.532,0.556)	0.011(-0.002,0.025)	0.088	0.234
LHR	0.526(0.514,0.538)	0.029(0.016,0.042)	< 0.001	0.001
SII	0.525(0.513,0.537)	0.031(0.018,0.044)	< 0.001	< 0.001
NLR	0.520(0.508,0.532)	0.036(0.023,0.049)	< 0.001	< 0.001
IBI	0.552(0.540,0.564)	0.004(-0.009,0.017)	0.581	0.667

*INFLA-score* Low-grade inflammation score, *CTI* C-reactive protein-triglyceride glucose index, *LHR* Lymphocyte-to-HDL ratio, *IBI* In-flammatory burden index, *NLR* Neutro-phil-to-lymphocyte ratio, *SII* Systemic-immune-inflammation index

### Characterization of the level of chronic low-grade inflammatory index in PCa patients

Maximum selection log-rank statistics identified an optimal threshold of 2 for INFLA in PCa patients (M = 9.1072, *p* < 0.001, Figure S1: Cut-off of INFLA-score in PCa patients). Based on this threshold, 4445 (33.76%) patients were identified as having a high INFLA-score. In the low- and high-risk groups, statistically significant differences were found when comparing different ages, BMI, ethnicity, years of education, Townsend deprivation index, testosterone, hypertension, diabetes mellitus, smoking, drinking status, and physical activity, all with *p* < 0.001 Characteristics of the patients’ levels of the Chronic Low-Grade Inflammation Index are shown in Table 3.

**Table 3:** Characteristics by level of INFLA score in patients with prostate cancer

Characteristic	INFLA score		
	Low Risk (*N* = 8721)	High Risk (*N* = 4445)	*P* value
White blood cells (10⁹/L), Median [Q1, Q3]	6.1 (5.3, 7.0)	8.0 (7.1, 9.1)	< 0.001
Platelets (10⁹/L), Median [Q1, Q3]	217.1 (189.0, 247.4)	266.0 (235.9, 300.00)	< 0.001
C-reactive protein (mg/L), Median [Q1, Q3]	1.0 (0.6, 1.7)	2.5 (1.4, 4.5)	< 0.001
Neutrophils (10⁹/L), Median [Q1, Q3]	3.6 (3.1, 4.2)	5.3 (4.6, 6.2)	< 0.001
Lymphocytes (10⁹/L), Median [Q1, Q3]	1.7 (1.4, 2.1)	1.8 (1.4, 2.2)	0.001
Age (years), Median [Q1, Q3]	63.0 (59.0, 66.0)	63.0 (60.0, 66.0)	< 0.001
Townsend deprivation index, Median [Q1, Q3]	-2.5 (-3.8, -0.3)	-2.3 (-3.7, 0.4)	< 0.001
Ethnicity, n (%)			< 0.001
White	8291 (95.1%)	4342 (97.7%)	
Black	245 (2.8%)	34 (0.8%)	
Asian	95 (1.1%)	36 (0.8%)	
Other	90 (1.0%)	33 (0.7%)	
Education (years), Median [Q1, Q3]	18.0 (15.0, 20.0)	16.0 (15.0, 20.0)	< 0.001
Smoking_status			< 0.001
Never	4447 (51.0%)	1811 (40.7%)	
Former	3719 (42.6%)	2019 (45.4%)	
Current	555 (6.4%)	615 (13.8%)	
Drinking_status			0.037
Never	203 (2.3%)	110 (2.5%)	
Former	243 (2.8%)	159 (3.6%)	
Current	8275 (94.9%)	4176 (93.9%)	
Body mass index (kg/m²), Median [Q1, Q3]	27.0 (24.8, 29.4)	27.8 (25.5, 30.5)	< 0.001
Physical activity, n (%)			< 0.001
Low	1448 (16.6%)	888 (20.0%)	
Moderate	3479 (39.9%)	1775 (39.9%)	
High	3794 (43.5%)	1782 (40.1%)	
History of prostate disease, n (%)			0.882
No	7481 (85.8%)	3818 (85.9%)	
Yes	1240 (14.2%)	627 (14.1%)	
Family history of prostate cancer, n (%)			0.440
No	7738 (88.7%)	3963 (89.2%)	
Yes	983 (11.3%)	482 (10.8%)	
Testosterone (nmol/L), Median [Q1, Q3]	11.7 (9.5, 14.2)	10.9 (8.8, 13.3)	< 0.001
Previous PSA screening, n (%)			0.105
Don’t know	334 (3.8%)	198 (4.4%)	
No	3639 (41.7%)	1892 (42.6%)	
Yes	4748 (54.4%)	2355 (53.0%)	
diabetes, n (%)			0.001
No	8211 (94.2%)	4117 (92.6%)	
Yes	510 (5.8%)	328 (7.4%)	
hypertension, n (%)			< 0.001
No	5534 (63.5%)	2395 (53.9%)	
Yes	3187 (36.5%)	2050 (46.1%)	
CVD, n (%)			0.098
No	8051 (92.3%)	4066 (91.5%)	
Yes	670 (7.7%)	379 (8.5%)	

### Kaplan–Meier survival analysis

**Fig. 1:**
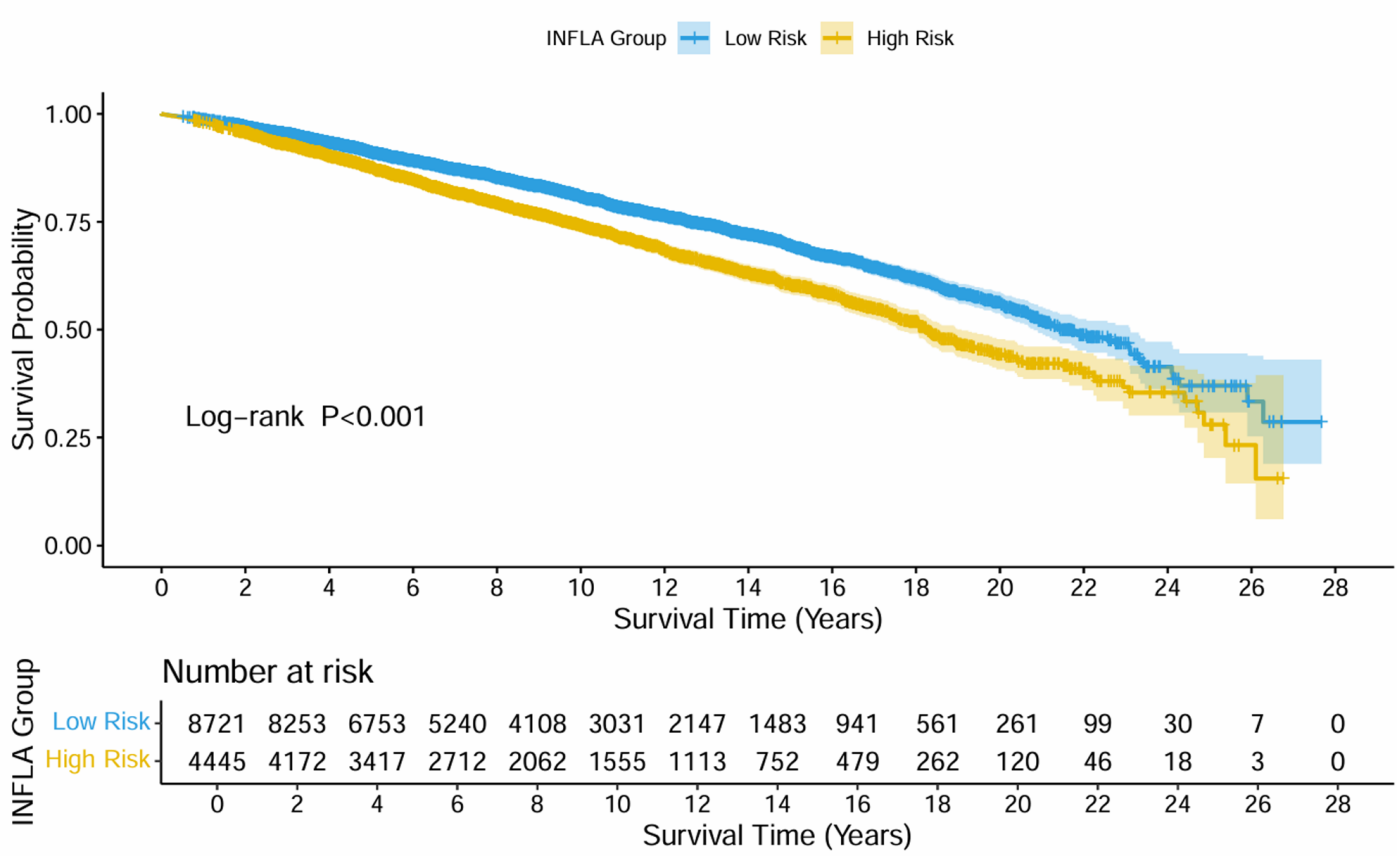
Kaplan–Meier curve of INFLA-score in patients with prostate cancer

We found that the INFLA-score could differentiate the prognosis of PCa patients (Fig. 1), with patients with a high INFLA-score having a significantly worse outcome than patients with a low INFLA-score (33.76% vs. 66.24%; log-rank *p* < 0.001).

### Univariate and multivariable survival analyses

**Fig. 2:**
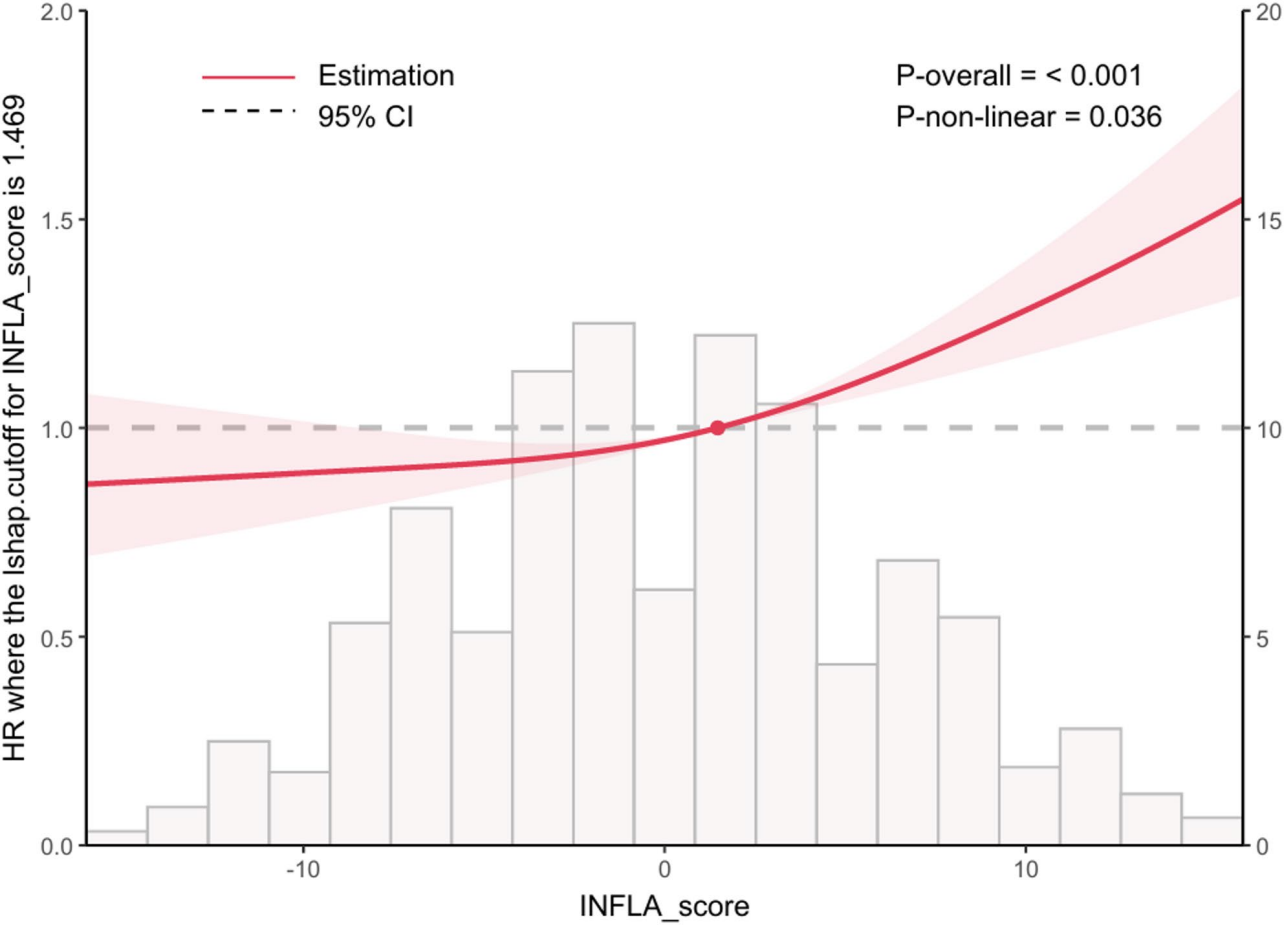
The association between low-grade inflammation score and overall survival in patients with prostate cancer. Adjusted for age, education, ethnic, Townsend Deprivation Index, Smoking, Drinking, BMI, Physical activity, History of prostate disease, Testosterone, Previous PSA screening, family history of PCa, diabetes, hypertension, CVD

Multivariate RCS curves showed a dose-response relationship between INFLA (continuous variable) and OS of PCa patients (Fig. 2). Multivariate Cox proportional risk regression analysis showed that INFLA-score as a continuous variable was an independent risk factor affecting the overall survival of PCa patients(HR 1.02, 95% CI 1.01–1.03, *p* < 0.01). When INFLA was assessed as a categorical variable (high vs. low), it was also an independent factor affecting the mortality risk of PCa patients (HR = 1.24, 95% CI: 1.15–1.33, *p* < 0.001). We further converted the INFLA score from a continuous variable to a categorical variable (quartiles) for Cox analysis, and using the Q1 group as a reference, the risk of mortality was progressively higher in the Q2, Q3, and Q4 groups, with HRs of 0.97, 1.15, and 1.27, respectively. In both the unadjusted crude model and the minimally corrected model, and in the fully adjusted model (model 3), this relationship showed significance ( *p* < 0.001), see Table 4.

**Table 4:** Association between INFLA-score and survival of patients with prostate cancer

INFLA	Model0	*P*	Model1	*P*	Model2	*P*
Continunous	1.03(1.03, 1.04)	< 0.001	1.03(1.02, 1.03)	< 0.001	1.02(1.01, 1.03)	< 0.001
Cut-off- value		< 0.001		< 0.001		< 0.001
C1(< 2)	Ref,		Ref,		Ref,	
C2(≥ 2)	1.41(1.31, 1.51)	< 0.001	1.35(1.25, 1.45)	< 0.001	1.24(1.15, 1.33)	< 0.001
Quartiles						
Q1	Ref,		Ref,		Ref,	
Q2	1.02(0.91, 1.14)	0.699	0.99(0.88, 1.11)	0.829	0.97(0.87, 1.09)	0.612
Q3	1.27(1.14, 1.42)	< 0.001	1.20(1.08, 1.34)	0.001	1.15(1.03, 1.28)	0.011
Q4	1.52(1.37, 1.68)	< 0.001	1.41(1.27, 1.57)	< 0.001	1.27(1.15, 1.42)	< 0.001
*P* for trend		< 0.001				< 0.001

Model 0: No adjusted. Model 1: Adjusted for age, education, ethnic, Townsend Deprivation Index. Model 2: Adjusted for age, education, ethnic, Townsend Deprivation Index, Smoking, Drinking, BMI, Physical activity, History of prostate disease, Testosterone, Previous PSA screening, family history of PCa, diabetes, hypertension, CVD

### Subgroup analysis

**Fig. 3:**
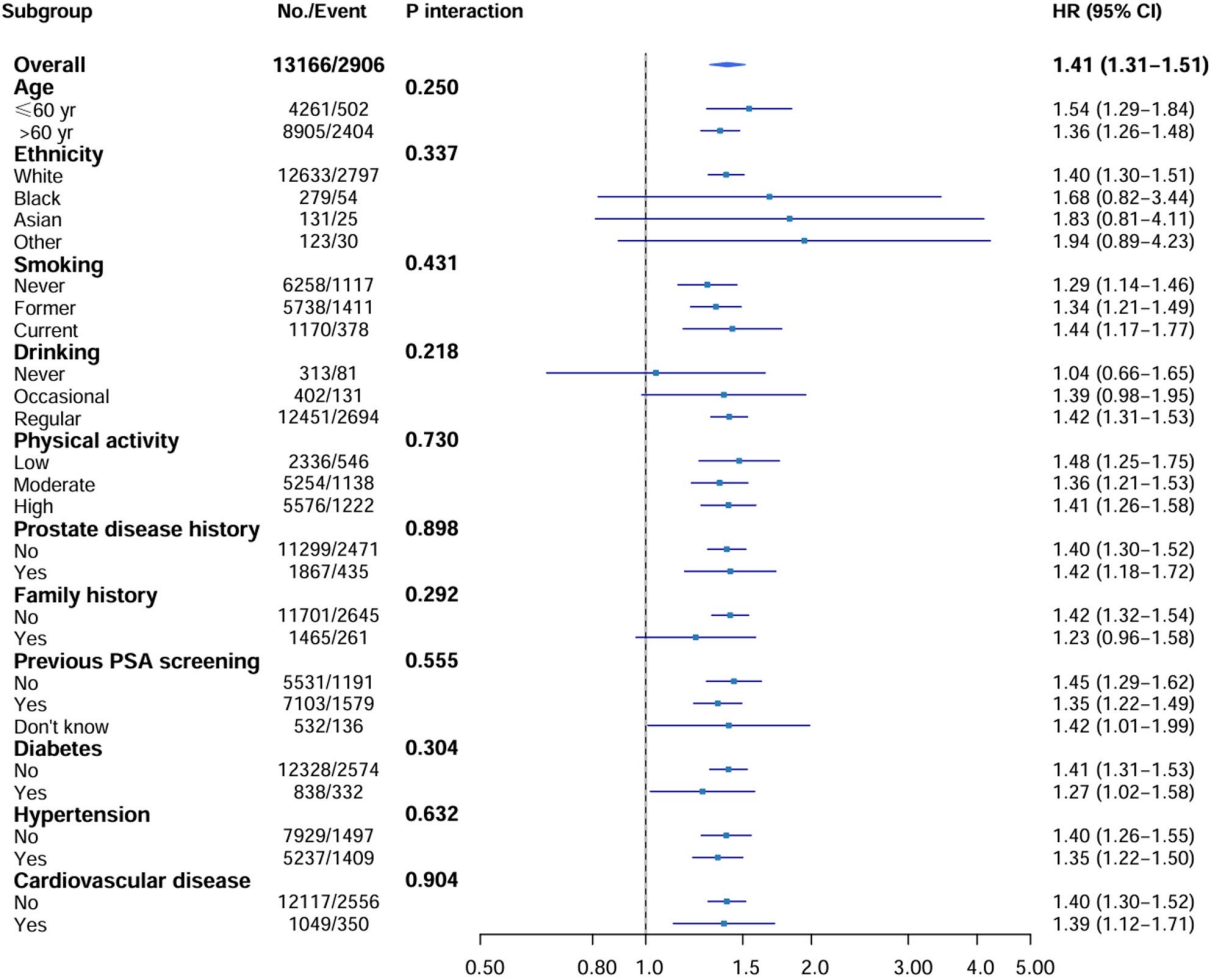
Subgroup and interaction analysis between INFLA-score and survival of patients with prostate cancer across various subgroups

Subgroup analysis showed that risk estimates for high INFLA-score were similar within subgroups(Fig. 3).

### Sensitivity analysis

To assess the robustness of the results of this study, we performed a series of sensitivity analyses. First, to assess potential reverse causality, we reran the analysis after excluding patients who died within the first two years of follow-up; the association between the INFLA-score and and increased all-cause mortality remained significant (HR = 1.24, 95% CI: 1.15–1.33, *p* < 0.001; Table S1: Association after excluding early deaths). Similarly, the results were consistent in a complete-case analysis excluding participants with missing covariate data (Table S2: Association in the complete-case cohort), and after excluding extreme outliers beyond mean ± 4 SD (Table S3: Association after removing extreme values).

## Discussion

Our analysis, based on a large prospective cohort, demonstrates that the INFLA-score is an independent predictor of overall survival (OS) in patients with prostate cancer (PCa). The study identified an optimal INFLA-score cut-off value of 2, above which a threshold indicating elevated levels of systemic inflammation was significantly associated with poor OS in PCa patients. These results suggest that the INFLA-score has good application value as a comprehensive inflammation assessment tool for risk stratification and survival prediction in PCa.

These findings are consistent with and extend the growing body of research on systemic inflammation within the UK Biobank cohort. While INFLA-score has been previously associated with various disease outcomes in the UK Biobank, including cardiovascular diseases and metabolic disorders [17, 20–23], our study is the first to specifically establish its prognostic value for prostate cancer survival. This cross-disease consistency reinforces the INFLA-score as a robust, generalizable marker of systemic inflammation with broad clinical applicability.

Similarly, our work aligns with and builds upon previous UK Biobank investigations examining inflammation-prostate cancer relationships. Prior studies have reported associations between individual inflammatory markers (such as C-reactive protein, leukocyte counts) or composite indices (like the systemic immune-inflammation index) and prostate cancer risk or progression [24–26]. By employing the INFLA-score—a multidimensional integration of multiple inflammatory pathways—we provide a more comprehensive assessment than single-parameter approaches. Importantly, our analysis extends beyond disease incidence to demonstrate that systemic inflammation, as captured by INFLA-score, independently predicts long-term survival in established prostate cancer cases.

The above findings provide new evidence for understanding the central role of systemic inflammation in tumor progression. Systemic inflammation, as an important component of the tumor microenvironment, plays a key role in influencing disease progression and outcomes in cancer patients [27, 28]. This is particularly relevant in PCa, where chronic inflammation is closely associated with the formation of precancerous lesions such as Proliferative Inflammatory Atrophy (PIA), which is considered to be one of the potential origins of prostate cancer [29]. These lesions are characterized by the morphological features of hyperplastic glandular epithelium with simple atrophy and are often associated with chronic inflammation [30]. In fact, the association between inflammation and tumors has a long history and consensus — it has been recognized as one of the key factors in cancer development, progression, and metastasis since the 19th century [31]; Trinchieri et al. [32]even categorized tumor-associated inflammation as hallmark of cancer of cancer, and uncontrolled inflammation is closely associated with tumorigenesis, progression, invasion and metastasis [33, 34]. Because of this [35], hematologic products of inflammatory processes can be considered as potential biomarkers.Although serum inflammatory biomarkers such as neutrophils, lymphocytes, and NLR have been shown to have prognostic value for survival in a variety of cancers, including PCa [36–38], there is still a gap in the studies that systematically compare the performance of different hematologic biomarkers in predicting mortality in PCa patients. To this end, we systematically evaluated and compared the prognostic value for OS of six inflammatory biomarkers based on peripheral blood parameters in patients with PCa. Our analysis identified the INFLA-score as the marker with the strongest association with overall survival, which suggests its potential utility for improving risk stratification and informing individualized treatment strategies.

The efficacy of the INFLA-score may stem from its multidimensional integration of inflammation and immunity. Compared to single indicators (such as the NLR, which reflects only the balance between granulocytes and lymphocytes) or other composite indices (such as the Systemic Immune-Inflammation Index (SII), which integrates only neutrophil, lymphocyte, and platelet counts), the INFLA-score uniquely incorporates the acute-phase protein CRP. This allows it to assess both the acute phase and chronic persistence of inflammation, thereby providing a more comprehensive depiction of the body’s systemic inflammatory landscape. As a non-specific inflammatory marker, CRP effectively reflects the level of inflammation during infection or tissue damage. By inducing the activation of endothelial cells and smooth muscle cells, it promotes the expression of adhesion molecules, chemokines, and vascular endothelial growth factor, thereby creating favorable conditions for tumor invasion [31]. Gómez-Gómez et al. found that pre-prostatectomy CRP levels were significantly associated with clinically significant PCa (csPCa) and higher Gleason scores [39]. In addition, persistent elevation of CRP is usually indicative of poor survival outcomes and risk of metastasis in cancer patients [34], and in metastatic PCa, elevated CRP is an even more independent predictor of poorer survival [40]. Neutrophils and lymphocytes, as important components of humoral immunity, play key roles in chemotaxis, phagocytosis, intracellular killing and adaptive immune regulation. Among them, lymphocytes are particularly important in anticancer immune responses in the circulating and tumor microenvironment, e.g. through T cell-mediated cytotoxicity [41]. Low lymphocyte counts may result in a weak, inadequate immune response to the tumor [42]. Studies have shown that elevated NLR correlates with higher Gleason scores in PCa [43], and neutrophil counts have been identified as a predictor of outcome in patients with limited prostate cancer (LPC) [44]. The effect of inflammatory response on leukocyte counts is usually manifested as neutrophilia and lymphopenia, and thus NLR is widely used in studies involving the diagnosis, staging, and prognosis of malignant tumors. It has been shown that in men with PSA levels of 4–10 ng/mL, elevated NLR is closely associated with the detection of PCa [45]. Several studies have also confirmed the relationship between the NLR and Gleason scores, including the application of the NLR to predict current Gleason scores and their subsequent elevation [46]. A meta-analysis by Gu et al. [47], encompassing both Western and Asian populations, further confirmed the prognostic value of the NLR for survival in PCa patients, and other studies have reached similar conclusions [48, 49]. In summary, multiple inflammatory indicators have shown important predictive value in PCa survival outcomes. As a comprehensive index integrating multiple inflammatory and immune parameters, the INFLA score can more systematically and comprehensively assess the inflammatory status of the body, and it shows significant advantages in predicting the survival of PCa patients, which has a broad clinical application prospect. It is suggested that the INFLA score should be more actively incorporated into the PCa risk assessment system in future studies to further validate its clinical utility and promotion value.

To our knowledge, this is the first study to comprehensively assess the prognostic value for OS of hematologic inflammatory biomarkers in patients with PCa. Unlike previous studies focusing on single indicators, we evaluated a clinical parameter combination, identifying the INFLA-score as optimal for predicting OS with a clinically applicable threshold. Using UK Biobank data enabled adjustment for numerous variables, enhancing reliability. However, there are some limitations to this study. First, although adjustments were made for multiple confounders, some variables were not considered, such as complex dietary patterns, family history of all cancers, and environmental exposure data (including environmental toxins and occupational exposures). Some data (e.g., physical activity levels) have missing values that may bias interpretation. Second, there is no information on tumor staging and grading. Therefore, we were unable to perform a comprehensive assessment of the association between biomarkers and cancer severity. In addition, due to the lack of data on other survival endpoints (e.g., tumor-specific survival, recurrence-free survival), this study was unable to explore the association between the INFLA score and these endpoints, which deserves subsequent in-depth study. Additionally, our study lacked data on prostate-specific antigen (PSA) levels, a key clinical biomarker for PCa. This limits our ability to assess the added value of the INFLA-score relative to standard PSA-based risk assessment. Furthermore, the threshold selection for the INFLA-score, while determined by established statistical methods and showing independent prognostic value, demonstrated variability in cross-validation analyses. This suggests the optimal cutoff may be influenced by sample characteristics and should be validated in independent cohorts. Finally, despite the large sample size and long follow-up in this study, the current follow-up duration may still be insufficient to capture long-term survival differences in prostate cancer given its slow progression. Future assessment through extended follow-up or or repeated measurements of inflammatory markers will help to better characterize the relationship between inflammation and prostate cancer survival.

## Conclusion

In conclusion, our study establishes the INFLA-score as the optimal systemic inflammatory biomarker for predicting OS in PCa, underscoring its value as an accessible tool for improving risk stratification and guiding personalized management. To advance its clinical translation, future research should prioritize external validation in diverse cohorts and investigate its association with cancer-specific endpoints and the prognostic significance of its dynamic changes. 

## Supplementary Information

Supplementary Material 1: Figure S1. Cut-off of INFLA-score in PCa patients. Figure S2. The association between CTI and overall survival in patients with prostate cancer. Figure S3. Association between inflammatory levels measured at different time periods before and after diagnosis and disease risk (Hazard Ratio). Figure S4. Meta analysis forest. Figure S5. Standardized Restricted Mean Survival Time (RMST) ratios for prostate cancer patients stratified by INFLA-score groups over 25-year follow-up. Table S1. Association between INFLA-score and survival of patients with prostate cancer after excluding patients who died within the first 2 years of follow-up. Table S2. Association between INFLA-score and survival of patients with prostate cancer after excluding participants with missing covariate data. Table S3. Association between INFLA-score and survival of patients with prostate cancer after excluding extreme outliers (beyond mean ± 4SD). Table S4. Interaction analysis between INFLA-score and time interval (pre-diagnosis measurements only). Table S5. Association between inflammatory markers and survival of patients with prostate cancer. Table S6. Model Comparison: Additional Prognostic Value of INFLA-score After Adjusting for All Covariates.Table S7. Five-year clinical outcomes by INFLA-score quartiles in prostate cancer patients.

## Data Availability

No original data are publicly available due to UK Biobank access protocols. This research was conducted under Application Number 540454. Bona fide researchers can apply for data access through the UK Biobank ([https://www.ukbiobank.ac.uk]).

## Abbreviations

BMI: Body Mass Index
CI: Confidence Interval
CRP: C-reactive Protein
HR: Hazard Ratio
INFLA-score: Low-grade Inflammation Score
NLR: Neutrophil-to-Lymphocyte Ratio
OS: Overall Survival
PCa: Prostate Cancer
PSA: Prostate-Specific Antigen
UKB: UK Biobank
WBC: White Blood Cell Count
