# Supplementary material for: The low-grade Inflammation Score (INFLA-Score) as a predictor of overall survival in prostate cancer: a UK biobank cohort study
Source: BMC Urol. 2026 May 8;26:156. doi: 10.1186/s12894-026-02078-5 (PMC13339364; doi:10.1186/s12894-026-02078-5)
Supplement: Supplementary file 1 — Supplementary Material 1: Figure S1. Cut-off of INFLA-score in PCa patients. Figure S2. The association between CTI and overall survival in patients with prostate cancer. Figure S3. Association between inflammatory levels measured at different time periods before and after diagnosis and disease risk (Hazard Ratio). Figure S4. Meta analysis forest. Figure S5. Standardized Restricted Mean Survival Time (RMST) ratios for prostate cancer patients stratified by INFLA-score groups over 25-year follow-up. Table S1. Association between INFLA-score and survival of patients with prostate cancer after excluding patients who died within the first 2 years of follow-up. Table S2. Association between INFLA-score and survival of patients with prostate cancer after excluding participants with missing covariate data. Table S3. Association between INFLA-score and survival of patients with prostate cancer after excluding extreme outliers (beyond mean ± 4SD). Table S4. Interaction analysis between INFLA-score and time interval (pre-diagnosis measurements only). Table S5. Association between inflammatory markers and survival of patients with prostate cancer. Table S6. Model Comparison: Additional Prognostic Value of INFLA-score After Adjusting for All Covariates.Table S7. Five-year clinical outcomes by INFLA-score quartiles in prostate cancer patients. [file 12894_2026_2078_MOESM1_ESM.docx]

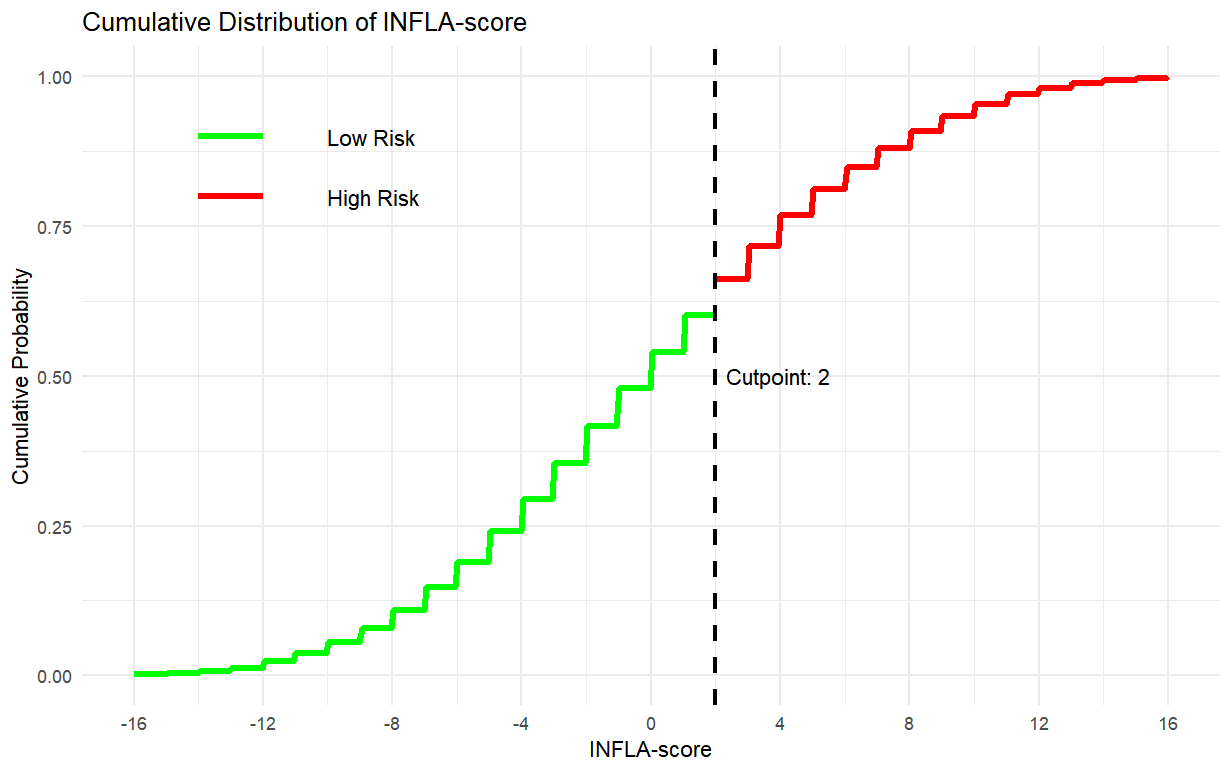


**Figure S1.** Cut-off of INFLA-score in PCa patients.


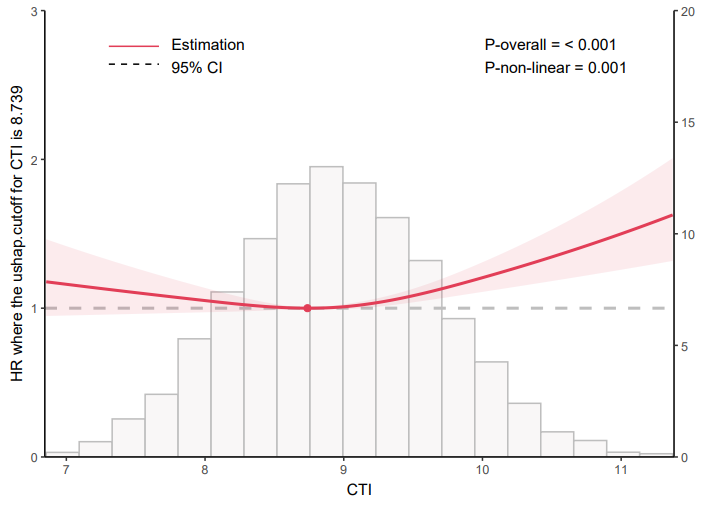


**Figure S2.** The association between CTI and overall survival in patients with prostate cancer. Adjusted for age, education, ethnic, Townsend Deprivation Index, Smoking, Drinking, BMI, Physical activity ,History of prostate disease, Testosterone, Previous PSA screening, family history of PCa, diabetes, hypertension, CVD.


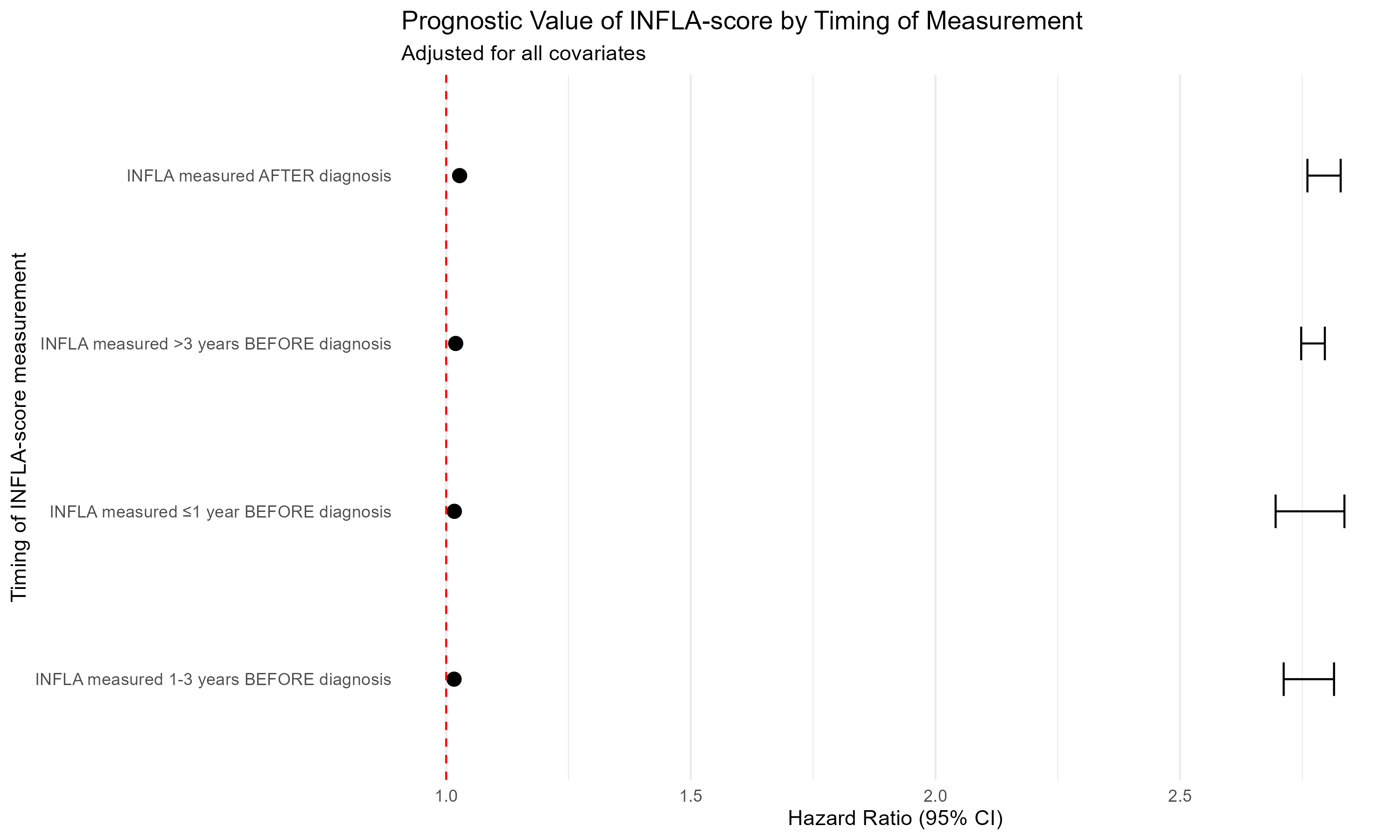


**Figure S3.**Association between inflammatory levels measured at different time periods before and after diagnosis and disease risk (Hazard Ratio)


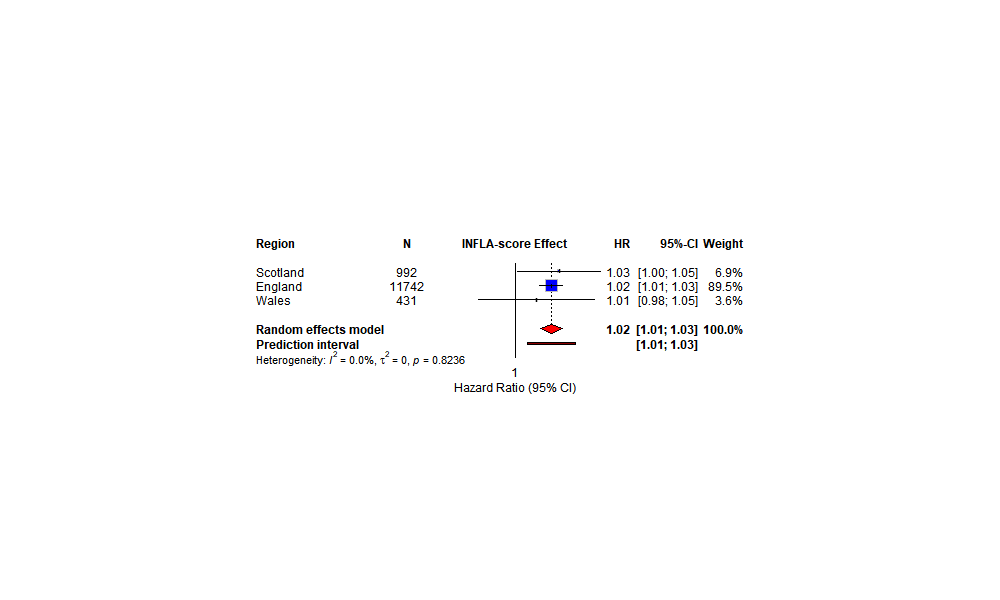


**Figure S4.**Meta analysis forest

****
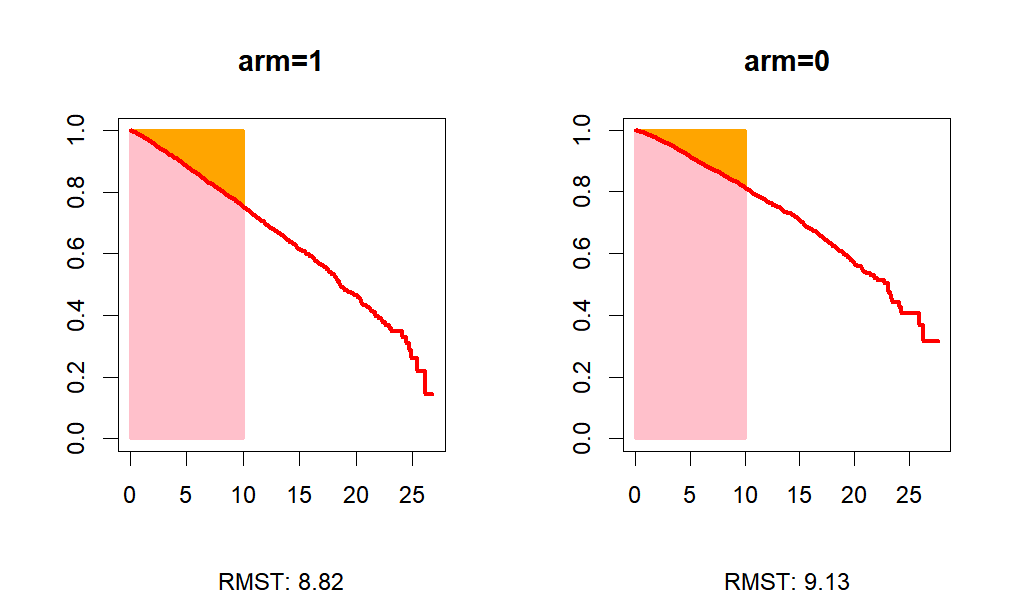
****

****Figure 5.**** Standardized Restricted Mean Survival Time (RMST) ratios for prostate cancer patients stratified by INFLA-score groups over 25-year follow-up. The x-axis represents time since diagnosis (years), and the y-axis represents the RMST ratio (arm=1 [High INFLA] vs. arm=0 [Low INFLA]). At time 0, both groups have a ratio of 1.00. Over time, the High INFLA group shows progressively lower survival probabilities compared to the Low INFLA group, with the difference becoming more pronounced with longer follow-up durations.

**Table S1.** Association between INFLA-score and survival of patients with prostate cancer after excluding patients who died within the first 2 years of follow-up.

| **INFLA** | **Model0** | ***P*** | **Model1** | ***P*** | **Model2** | ***P*** |
| --- | --- | --- | --- | --- | --- | --- |
| Continunous | 1.03(1.03, 1.04) | <0.001 | 1.03(1.02, 1.03) | <0.001 | 1.02(1.01, 1.03) | <0.001 |
| Cut-off- value |  | <0.001 |  | <0.001 |  | <0.001 |
| C1(<2) | Ref, |  | Ref, |  | Ref, |  |
| C2(≥2) | 1.41(1.31, 1.51) | <0.001 | 1.34(1.25, 1.45) | <0.001 | 1.24(1.15, 1.33) | <0.001 |
| Quartiles |  |  |  |  |  |  |
| Q1 | Ref, |  | Ref, |  | Ref, |  |
| Q2 | 1.02(0.91, 1.14) | 0.699 | 0.99(0.88, 1.11) | 0.828 | 0.97(0.87-1.09) | 0.599 |
| Q3 | 1.27(1.14, 1.42) | <0.001 | 1.20(1.08, 1.34) | 0.001 | 1.15(1.03, 1.28) | 0.011 |
| Q4 | 1.52(1.37, 1.68) | <0.001 | 1.41(1.27, 1.56) | <0.001 | 1.27(1.14-1.41) | <0.001 |
| P for trend |  | <0.001 |  |  |  | <0.001 |

Model 0: No adjusted. Model 1: Adjusted for age, education, ethnic, Townsend Deprivation Index. Model 2: Adjusted for age, education, ethnic, Townsend Deprivation Index, Smoking, Drinking, BMI, Physical activity ,History of prostate disease, Testosterone, Previous PSA screening, family history of PCa, diabetes, hypertension, CVD.

**Table S2.** Association between INFLA-score and survival of patients with prostate cancer after excluding participants with missing covariate data.

| **INFLA** | **Model0** | ***P*** | **Model1** | ***P*** | **Model2** | ***P*** |
| --- | --- | --- | --- | --- | --- | --- |
| Continunous | 1.03(1.02, 1.04) | <0.001 | 1.02(1.02, 1.03) | <0.001 | 1.02(1.01, 1.03) | <0.001 |
| Cut-off- value |  | <0.001 |  | <0.001 |  | <0.001 |
| C1(<2) | Ref, |  | Ref, |  | Ref, |  |
| C2(≥2) | 1.38(1.26, 1.50) | <0.001 | 1.32(1.21, 1.44) | <0.001 | 1.19(1.09, 1.30) | <0.001 |
| Quartiles |  |  |  |  |  |  |
| Q1 | Ref, |  | Ref, |  | Ref, |  |
| Q2 | 1.01(0.89, 1.15) | 0.868 | 0.98(0.85, 1.11) | 0.709 | 0.95(0.84-1.09) | 0.494 |
| Q3 | 1.29(1.14, 1.46) | <0.001 | 1.21(1.07, 1.38) | 0.003 | 1.15(1.01, 1.31) | 0.031 |
| Q4 | 1.46(1.29, 1.64) | <0.001 | 1.35(1.20, 1.53) | <0.001 | 1.20(1.06-1.36) | 0.005 |
| P for trend |  | <0.001 |  |  |  | <0.001 |

Model 0: No adjusted. Model 1: Adjusted for age, education, ethnic, Townsend Deprivation Index. Model 2: Adjusted for age, education, ethnic, Townsend Deprivation Index, Smoking, Drinking, BMI, Physical activity ,History of prostate disease, Testosterone, Previous PSA screening, family history of PCa, diabetes, hypertension, CVD.

**Table S3.** Association between INFLA-score and survival of patients with prostate cancer after excluding extreme outliers (beyond mean ± 4SD).

| **INFLA** | **Model0** | ***P*** | **Model1** | ***P*** | **Model2** | ***P*** |
| --- | --- | --- | --- | --- | --- | --- |
| Continunous | 1.03(1.02, 1.04) | <0.001 | 1.03(1.02, 1.03) | <0.001 | 1.02(1.01, 1.03) | <0.001 |
| Cut-off- value |  | <0.001 |  | <0.001 |  | <0.001 |
| C1(<2) | Ref, |  | Ref, |  | Ref, |  |
| C2(≥2) | 1.38(1.28, 1.49) | <0.001 | 1.32(1.23, 1.43) | <0.001 | 1.23(1.14, 1.33) | <0.001 |
| Quartiles |  |  |  |  |  |  |
| Q1 | Ref, |  | Ref, |  | Ref, |  |
| Q2 | 1.04(0.93, 1.17) | 0.451 | 1.01(0.90, 1.13) | 0.888 | 1.00(0.90-1.13) | 0.941 |
| Q3 | 1.27(1.14, 1.41) | <0.001 | 1.20(1.07, 1.34) | 0.001 | 1.15(1.03, 1.28) | 0.014 |
| Q4 | 1.49(1.35, 1.66) | <0.001 | 1.39(1.25, 1.55) | <0.001 | 1.28(1.15-1.43) | <0.001 |
| P for trend |  | <0.001 |  |  |  | <0.001 |

Model 0: No adjusted. Model 1: Adjusted for age, education, ethnic, Townsend Deprivation Index. Model 2: Adjusted for age, education, ethnic, Townsend Deprivation Index, Smoking, Drinking, BMI, Physical activity ,History of prostate disease, Testosterone, Previous PSA screening, family history of PCa, diabetes, hypertension, CVD.

**Table S4** Interaction analysis between INFLA-score and time interval (pre-diagnosis measurements only)

|  | **β（SE）** | **HR** | ***P*** |
| --- | --- | --- | --- |
| Main effect |  |  |  |
| INFLA-score(per1-unit increase) | 0.0242 (0.0072) | 1.0245（1.0104-1.0389） | <0.001 |
| Time from baseline to diagnosis (per1-year increase) | 0.1849 (0.0093) | 1.203（1.1842-1.2220） | <0.001 |
| Interaction |  |  |  |
| INFLA-score × Time interval | -0.0010 (0.0011) | 0.9990（0.9969-1.0012） | 0.3644 |

Interaction analysis between INFLA-score and time from baseline measurement to prostate cancer diagnosis, using only pre-diagnosis measurements (n=10,368). The Cox proportional hazards model included both main effects and their interaction term, adjusted for age, ethnicity, Townsend deprivation index, education, smoking status, drinking status, BMI, physical activity, history of PSA testing, testosterone levels, history of prostate disease, family history of prostate cancer, hypertension, diabetes, and cardiovascular disease. The interaction term was not statistically significant (P=0.364), indicating that the association between INFLA-score and all-cause mortality does not vary significantly with the time interval between baseline measurement and cancer diagnosis.

**Table S5** Association between inflammatory markers and survival of patients with prostate cancer.

|  | **Model0** | ***P*** | **Model1** | ***P*** | **Model2** | ***P*** |
| --- | --- | --- | --- | --- | --- | --- |
| IBI Continunous | 1.00(1.00, 1.00) | <0.001 | 1.00(1.00, 1.00) | <0.001 | 1.00(1.00, 1.00) | <0.001 |
| Q1 | Ref, |  | Ref, |  | Ref, |  |
| Q2 | 1.02(0.91, 1.14) | 0.699 | 0.99(0.88, 1.11) | 0.829 | 0.97(0.87, 1.09) | 0.612 |
| Q3 | 1.27(1.14, 1.42) | <0.001 | 1.20(1.08, 1.34) | 0.001 | 1.15(1.03, 1.28) | 0.011 |
| Q4 | 1.52(1.37, 1.68) | <0.001 | 1.41(1.27, 1.57) | <0.001 | 1.27(1.15-1.42) | <0.001 |
| P for trend |  | <0.001 |  |  |  | <0.001 |
| CTI  Continunous | 1.25(1.19,1.31） | <0.001 | 1.21 (1.15, 1.28） |  | 1.10 (1.05, 1.17) | <0.001 |
| Q1 |  |  |  |  |  |  |
| Q2 | 1.04(0.93, 1.16) | 0.494 | 1.03 (0.92-1.15) | 0.594 | 0.95 (0.85, 1.06） | 0.377 |
| Q3 | 1.19(1.07, 1.32) | 0.002 | 1.16 (1.04, 1.29) | 0.008 | 1.01 (0.90, 1.13） | 0.871 |
| Q4 | 1.47(1.33, 1.63) | <0.001 | 1.40 (1.27, 1.56) | <0.001 | 1.16 (1.03, 1.2） | 0.012 |
| P for trend |  | <0.001 |  |  |  | <0.001 |

Model 0: No adjusted. Model 1: Adjusted for age, education, ethnic, Townsend Deprivation Index. Model 2: Adjusted for age, education, ethnic, Townsend Deprivation Index, Smoking, Drinking, BMI, Physical activity, History of prostate disease, Testosterone, Previous PSA screening, family history of PCa, diabetes, hypertension, CVD.

**Table S6**. Model Comparison: Additional Prognostic Value of INFLA-score After Adjusting for All Covariates

|  | **Covariates-Only Model** | **Covariates+ INFLA Model** | **Change/Improvement** | ***P*** |
| --- | --- | --- | --- | --- |
| Likelihood Ratio Test | - | - | χ²= 40.37 | <0.001 |
| AIC | 49,521.62 | 49,483.25 | ΔAIC = -38.37 | - |
| Nagelkerke's Pseudo R² | 0.0692 | 0.0721 | ΔR² = +0.0029 | - |
| Harrell's C-index | ΔR² = +0.0029 | 0.3351 | -0.0031 | - |

Notes:AIC = Akaike Information Criterion; ΔAIC = change in AIC; ΔR² = change in R-squared.

A lower AIC indicates better model fit. The likelihood ratio test χ² statistic tests whether adding INFLA-score significantly improves the model over covariates alone.

**Table S7**. Five-year clinical outcomes by INFLA-score quartiles in prostate cancer patients

| INFLA-score Quartile | **No. Of Patients** | **5-Year Survival(%)** | **5-Year Mortality (%)** | **Relative Risk (vs Q1)** |
| --- | --- | --- | --- | --- |
| Q1 (Lowest) | 3881 | 91.6 | 8.4 | 1.00 (Ref) |
| Q2 | 3,232 | 90.9 | 9.1 | 1.08 |
| Q3 | 2,999 | 89.6 | 10.4 | 1.24 |
| Q4(Highest) | 3,053 | 87.1 | 12.9 | 1.54 |
